# Supplementary material for: Predicting protein-peptide interaction sites using distant protein complexes as structural templates
Source: Sci Rep. 2019 Mar 12;9:4267. doi: 10.1038/s41598-019-38498-7 (PMC6414505; doi:10.1038/s41598-019-38498-7)
Supplement: Supplementary file 1 — Supplementary Info [file 41598_2019_38498_MOESM1_ESM.pdf]

Supplementary Information for "Predicting protein-peptide interaction sites using distant protein complexes as structural templates"

Isak Johansson-Åkhe[1] Claudio Mirabello[1] and Björn Wallner[1,*]

December 10, 2018

1 Description of Features

Below, the features used by the Random Forest in InterPep are described in more detail. Two residues are defined as interacting if their closest heavy atoms are within 6Å.

Length: Five features relate to different lengths:

1. Length of the target peptide.
2. Length of the target protein chain.
3. Length of the aligned partner chain.
4. Length of the aligned segment.
5. Number of residues in the aligned segment that are involved in inter-chain interaction in the aligned partner chain, i.e the length of the proposed interaction surface.

Alignment Quality: Three features are based on the quality of the structural alignment:

1. TM-score normalized on the target protein chain.
2. TM-score normalized on the aligned partner chain.
3. RMSD of the alignment.

Aligned Region Complexity: Small and simple motifs will score a large number of structural hits that might not be relevant.

1. Contact order of aligned region of the target chain.
2. Length of the longest aligned α-helix.

[1]Division of Bioinformatics, Department of Physics, Chemistry and Biology, Linköping University, SE-581 83 Linköping, Sweden

*To whom correspondence should be addressed: bjorn.wallner@liu.se

Amino Acid Composition Distance: These features are included to measure the similarity in amino acid composition between the targets and templates, as amino acid composition directly influences the likelihood of interaction [6, 7]. They are produced in several steps. First, for each surface (or peptide) a composition vector 20 values long is calculated, each element being a percentile value representing the fraction of the surface which is made up of that amino acid. Then, the BLOSUM62 matrix is multiplied by this vector to produce a new vector not representative of the exact composition of the surface, but rather its "compositional space":

$$\vec{y}_i = B\vec{x}_i \tag{1}$$

where $\vec{y}_i$ is the compositional space vector of the surface i, B is the BLOSUM62 matrix, and $\vec{x}_i$ is the amino acid composition vector of the surface i. The Amino Acid Composition Difference between two surfaces j and k is the angle θ between the compositional space vectors $\vec{y}_j$ and $\vec{y}_k$:

$$\theta_{jk} = \arccos\left(\frac{\vec{y}_j \cdot \vec{y}_k}{\|\vec{y}_j\| + \|\vec{y}_k\|}\right) \tag{2}$$

1. Between the target peptide sequence and the residues of the template which the aligned parts of the template structure interact with, i.e the peptide representative part of the template.
2. Between the residues of the target chain that have aligned counterparts involved in protein-protein interaction, i.e the proposed interaction surface, and the residues these align to in the template structure.

Secondary Structure: Two vectors of three values each represent the relative secondary structure composition for:

1. Predicted secondary structure of the peptide using PSIPRED [5].
2. Actual secondary structure defined by STRIDE [3] for the residues interacting with the target protein in the template.

Surface Information: These represent additional information regarding the proposed interaction surface.

1. Relative exposure of the residues of the proposed interaction surface, measured with NACCESS [4]. Completely buried residues should be less likely to be involved in interactions.
2. Mean relative conservation of the residues of the proposed interaction surface, calculated with PSIBLAST [1]. Higher conservation indicates the residues are vital to protein function [2].

Peptide Template Information: Three values give information about the parts of chains in the aligned partner's file which interact with it. These features were included to measure how likely it is that the surface can be replaced by a single, small, and uninterrupted peptide chain.

1. Mean sequential distance between interacting residues. The distance was capped at 10 residues. If multiple chains are involved, the sequential difference between them counts as 10 residues.
2. Median sequential distance between interacting residues. The distance was capped at 10 residues. If multiple chains are involved, the sequential difference between them counts as 10 residues.
3. Summed lengths of all chains interacting with the aligned partner chain.

Model Information (optional): The sequence identity between the target and the template used in the modeling step. This feature is only used if the target structure is modeled.

2 Performance of Random Forest

2.1 Calculating Gini Impurity

Gini Impurity is used both for training and analyzing decision trees, as from a Random Forest.

At any given point in a decision tree, the Gini Impurity is the probability of incorrectly assigning a label to a target if labels would be assigned randomly by the distribution of labels at that point. In the case of two possible labels (False or True, 0 or 1), the total Gini impurity for a node is calculated as follows:

$$G(x) = 1 - \sum_{i=0}^{1} p_i^2 = 1 - p_0^2 - p_1^2 \tag{3}$$

where $G(x)$ is the Gini impurity of node x, and p_i is the probability that the label i is chosen if randomly sampling the incoming population.

The importance $Q(f)$ of a feature f in a decision tree is measured by the total reduction in Gini impurity over all branches split on that feature, as seen in equation 4. In the case of calculating the total importance of a group of features, branchings which lead to further branchings on a feature from the group are disregarded.

$$Q(f) = \sum_{i \in k(f)} (n_i * G_i - n_{ir} * G_{ir} - n_{il} * G_{il}) \tag{4}$$

where $k(f)$ is a list of all branches split on the feature f, i denotes a specific branch ($i \in k(f)$), n_x is the total number of samples from the training set which arrived at branch x, G_x is the Gini impurity of branch x, ir denotes the right child node of branch i, and il denotes the left child node of branch i. To get the relative feature importance for each feature, $Q(f)$ is normalized by the total importance of all features.

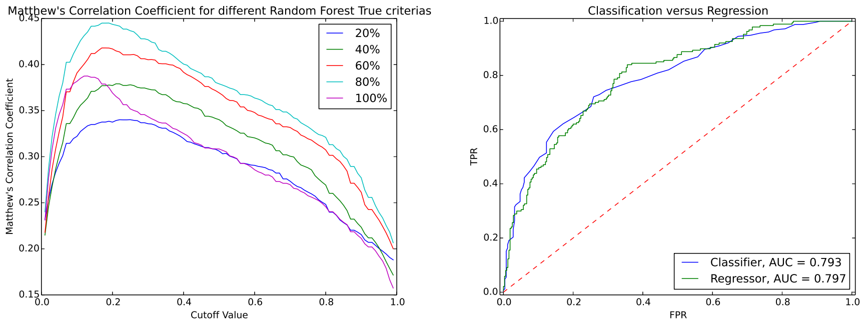

(a) (b)

Figure 1: **(a)**: Matthew's correlation coefficient for final local score residue predictions of InterPep with different score cutoffs for what is considered a true prediction of a residue. Note that this is not the mean Matthew's correlation coefficient over the proteins, but rather the correlation over all residues in the data-set. **(b)**: ROC-curve of the performance of the Random Forest Classifier as compared to the Random Forest Regressor.

2.2 Optimizing the Random Forest

Different parameters were tested for the Random Forest Classifier.

Firstly, the PPV cutoff for deciding if a template interaction site is regarded as "correct" or not was varied from 0.2 to 1.0. Additionally, a test was conducted where the Random Forest Classifier was substituted for a Random Forest Regressor. Results shown in figure 1. Note that from (a), we can also deduce that the optimal cutoff for residue scoring is 0.19. From these figures, it was clear that if using a classifier, the precision cutoff for evaluating a site as correct should be 80%. However, it was not clear if a Regressor should be used instead. When looking at the total number of correctly identified peptide-binding sites however, the Regressor found only 244, as compared to the Classifier finding 255. Thus, InterPep uses a Random Forest Classifier.

2.3 Performance for Model Structures

As different Random Forests were required for the native data-set and the modeled data-set, there should be some difference in how the features are treated, figure 2. However, as can be seen in the figure, the differences were all minor and well within standard deviation, except for the case of the new feature of sequence identity. This means that either this new feature alone stands for most of the difference, or the other features simply kept their importance but received different split-values in their branchings.

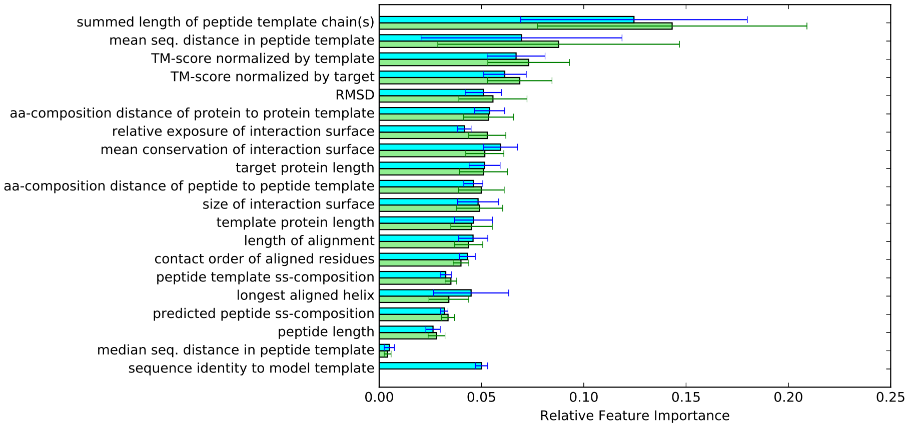

Figure 2: Comparison of relative feature importance between the Random Forest trained on native structures (shown in green), and the one trained on both native and modeled structure (shown in blue).

3 InterPep score

Figures describing the correlation between Matthew's correlation coefficient and InterPep score can be found in figure 3. From these it can be noted that the results should not be trusted for InterPep score less than 0.5, can be seen as ambiguous around 0.6, and can be trusted with few errors at 0.8 or above.

4 Models

InterPepM for models has comparable performance with InterPep for native structures. As such, if InterPep fails, perhaps generating a model for a native structure and running InterPepM could find the correct answer. In Figure 4, the results from several models are taken into consideration, and the result from the model with the highest InterPep global score was chosen to represent the final prediction for the target modeled. As is apparent however, the performance increase is negligible, as performance starts at 255 correctly identified sites and ends at 279, when considering 2 modeled structures in addition to the native one.

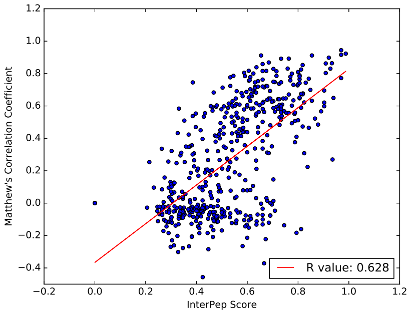

(a)

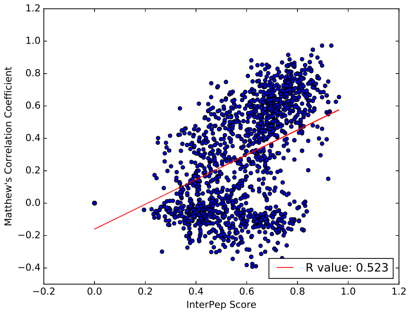

(b)

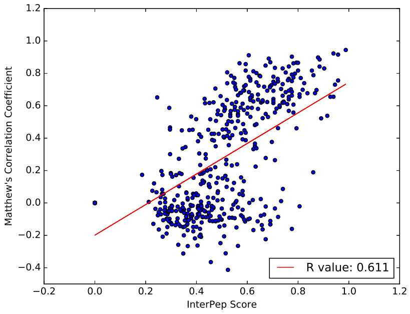

(c)

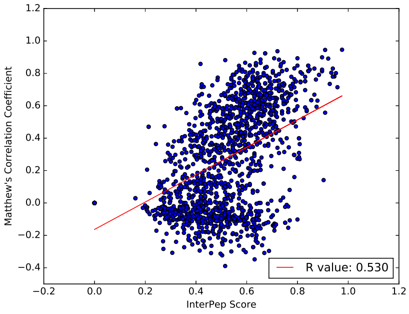

(d)

Figure 3: Correlation between InterPep global score and Matthew's correlation coefficient between local score and native contacts for predictions on **(a and c)**: native structures, and **(b and d)**: modeled structures. In **(a and b)**: the standard InterPep was used. In **(c and d)**: InterPepM was used, which is the same as InterPep except trained on both model and native structures, and with an extra feature for the sequence identity from the modeling.

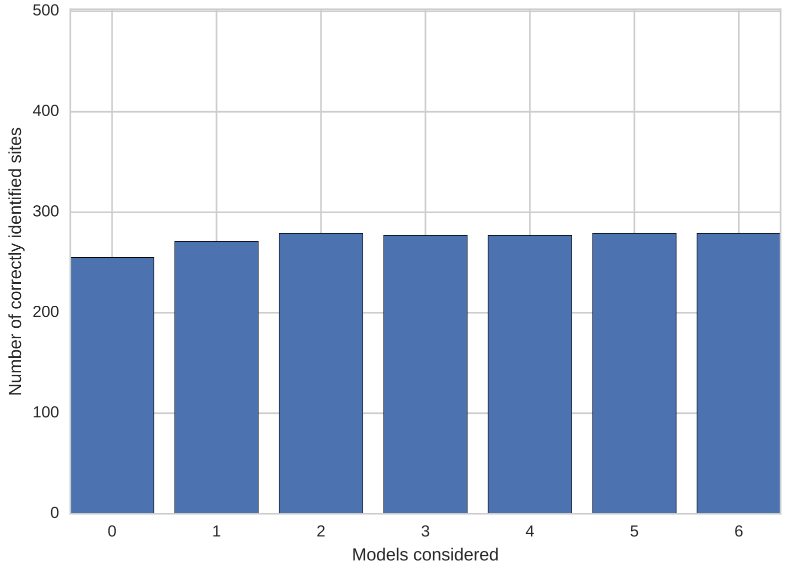

Figure 4: Performance increase from choosing the highest scoring result from several models, rather than just the model with best sequence identity. The first bar represents only running InterPep on the native structure, and not considering any models.

References

[1] Stephen F Altschul, Thomas L Madden, Alejandro A Schäffer, Jinghui Zhang, Zheng Zhang, Webb Miller, and David J Lipman. Gapped BLAST and PSI-BLAST: a new generation of protein database search programs. *Nucleic Acids Research*, 25(17):3389–3402, 1997.

[2] Daniel R Caffrey, Shyamal Somaroo, Jason D Hughes, Julian Mintseris, and Enoch S Huang. Are protein–protein interfaces more conserved in sequence than the rest of the protein surface? *Protein Science*, 13(1):190–202, 2004.

[3] Dmitrij Frishman and Patrick Argos. Knowledge-based protein secondary structure assignment. *Proteins: Structure, Function, and Bioinformatics*, 23(4):566–579, 1995.

[4] Simon J Hubbard and Janet M Thornton. Naccess. *Computer Program, Department of Biochemistry and Molecular Biology, University College London*, 2(1), 1993.

[5] David T Jones. Protein secondary structure prediction based on position-specific scoring matrices. *Journal of Molecular Biology*, 292(2):195–202, 1999.

[6] Nickolay A Khazanov and Heather A Carlson. Exploring the composition of protein-ligand binding sites on a large scale. *PLoS Comput Biol*, 9(11):e1003321, 2013.

[7] Gregory A Petsko and Dagmar Ringe. *Protein structure and function*. New Science Press, 2004.
